# A109 A COMPARISON OF MULTIMORBIDITY AT DEATH AMONG PERSONS WITH AND WITHOUT INFLAMMATORY BOWEL DISEASE: A POPULATION-BASED STUDY

**DOI:** 10.1093/jcag/gwad061.109

**Published:** 2024-02-14

**Authors:** G Postill, F Tang, E Kuenzig, E Buajitti, V Harish, E I Benchimol

**Affiliations:** University of Toronto Temerty Faculty of Medicine, Toronto, ON, Canada; SickKids Research Institute, Toronto, ON, Canada; SickKids Research Institute, Toronto, ON, Canada; University of Toronto Dalla Lana School of Public Health, Toronto, ON, Canada; University of Toronto Temerty Faculty of Medicine, Toronto, ON, Canada; SickKids Research Institute, Toronto, ON, Canada

## Abstract

**Background:**

With improved care and life-expectancy, people with IBD are living longer and more likely to develop multiple chronic conditions. However, research on co-morbidities in the IBD population has typically focused on co-occurrences of a specific disease alongside IBD and employed a cross-sectional or short-term cohort perspective. To date, no work has taken a life-course perspective, evaluating retrospectively from death, to identify the population-level burden of multimorbidity.

**Aims:**

To identify the burden of chronic conditions at time of death among people with IBD relative to their non-IBD counterparts.

**Methods:**

We conducted a retrospective matched cohort study using health administrative data from Ontario, Canada. Individuals with IBD (identified using a validated algorithm) who died between 2010 to 2020 were matched to controls (1:5 ratio) based on sex, age at death, and year of death. We calculated the proportion of decedents with each of 17 different chronic conditions and the number of conditions at death. Conditions were identified using validated algorithms or clusters of diagnostic codes (when validated algorithms were not available). The number of chronic conditions at death among people with and without IBD was modeled using Poisson regression adjusting for Ontario Marginalization Index and accounting for matching.

**Results:**

At the time of death, people with IBD (n=9728) were more likely than matched controls (n=42,389) to have mood disorders (69.0% vs. 60.2%), asthma (21.4% vs. 16.5%), renal failure (49.6% vs. 38.5%), osteoarthritis (76.8 vs. 67.6%), and osteoporosis (21.2% vs. 12.8%) (Figure 1B). Compared to their controls, people with IBD had were more likely to have ≥5 conditions (77.8% vs. 70.1%) (Figure 1A). People with IBD had significantly more chronic health conditions (RR 1.11, 95% CI 1.10-1.12).

**Conclusions:**

People with IBD accumulate a greater degree of morbidity compared to those without IBD. Our findings add a novel understanding of multimorbidity among those with IBD from a life-course perspective. Our research highlights the need to provide quality multi-disciplinary care to people with IBD across the life course to address the increased frequency of multimorbidity.

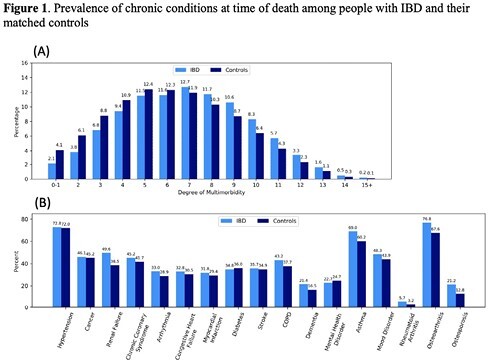

**Funding Agencies:**

ACG

